# Potential immune escape mechanisms underlying the distinct clinical outcome of immune checkpoint blockades in small cell lung cancer

**DOI:** 10.1186/s13045-019-0753-2

**Published:** 2019-06-28

**Authors:** Yaru Tian, Xiaoyang Zhai, Anqin Han, Hui Zhu, Jinming Yu

**Affiliations:** 1 Department of Radiation Oncology, Shandong Cancer Hospital and Institute, Shandong University, Jinan, China; 2 Department of Radiation Oncology, Shandong First Medical University and Shandong Academy of Medical Sciences, Jinan, China; 3 Department of Radiation Oncology, Shandong Cancer Hospital and Institute, Shandong First Medical University and Shandong Academy of Medical Sciences, 440 Jiyan Road, Jinan, 250117 China

**Keywords:** SCLC, Immune checkpoint blockades, Immune escape mechanisms

## Abstract

Small cell lung cancer (SCLC) is one of the deadliest cancer types in the world. Despite the high response rate to frontline platinum-containing doublets, relapse is inevitable for the majority of patients and the prognosis is poor. Topotecan, which has limited efficacy, has remained the standard second-line therapy for approximately three decades. Although SCLC has a high mutation burden, the clinical efficacy of immune checkpoint blockades (ICBs) in SCLC is far less pronounced than that in non-small cell lung cancer (NSCLC). Only atezolizumab in combination with chemotherapy improved overall survival over chemotherapy alone in the phase III CheckMate 133 trial and has recently received FDA approval as first-line therapy. Most studies concerning ICBs in SCLC are limited to early-phase studies and found that ICBs were not superior to traditional chemotherapy. Why is there such a large difference between SCLC and NSCLC? In this review, comparative analyses of previous studies indicate that SCLC is even more immunodeficient than NSCLC and the potential immune escape mechanisms in SCLC may involve the low expression of PD-L1 and the downregulation of major histocompability complex (MHC) molecules and regulatory chemokines. In consideration of these immune dysfunctions, we speculate that chemotherapy and radiotherapy prior to immunotherapy, the combination of ICBs with antiangiogenic treatment, and selecting tumor mutation burden in combination with PD-L1 expression as biomarkers could be promising strategies to improve the clinical efficacy of immunotherapy for SCLC.

## Background

Worldwide, lung cancer is the leading cause of cancer incidence and mortality, with 2.1 million new cases and 1.8 million deaths estimated in 2018, representing 18.4% of the total cancer-related deaths [1]. Small cell lung cancer (SCLC) accounts for approximately 14% of all lung cancers [2, 3] and is highly aggressive and lethal, characterized by a rapid growth rate and early development of widespread metastasis. Most patients with SCLC have a history of cigarette smoking [4–6] and harbor a high prevalence of somatic mutations [7–10]. SCLC is classified as limited-stage SCLC (LS-SCLC) and extensive-stage SCLC (ES-SCLC). ES-SCLC, generally with distant metastasis at the time of diagnosis, accounts for approximately two thirds of all SCLC. Platinum-based chemotherapy remains the first-line standard of care for SCLC and the response rate could reach more than 70%. Although SCLC is highly responsive to initial therapy, most patients with SCLC will inevitably relapse. Consequently, the prognosis of SCLC is rather poor, with an estimated 2-year overall survival (OS) of less than 5% [11–15]. Currently, topotecan is the only drug approved by the US Food and Drug Administration (FDA) as a second-line therapy. However, the efficacy of topotecan is limited, with a response rate of approximately 25% for platinum-sensitive patients and less than 10% for platinum-resistant or refractory patients [16–18]. Other recommended second-line options included irinotecan, vinorelbine, gemcitabine, and temozolomide [11]. Clinical benefit is even dismal for treatment beyond the second line. Contrasting sharply with non-small cell lung cancer (NSCLC), SCLC is a hard-to-treat cancer with lack of progress for nearly 30 years.

Immunotherapy has revolutionized the standard of care across multiple cancer types. As tumors develop, they can escape immune surveillance by various mechanisms in steps in the cancer-immunity cycle [19, 20]. Immune checkpoints, limiting antitumor responses and contributing to immune escape, have been readily confirmed as negative regulators in recent years. Two such checkpoints, cytotoxic T-lymphocyte protein 4 (CTLA4) and programmed cell death protein-1 (PD-1), are the best [21]. The co-inhibitory receptor CTLA-4, which is expressed on T cells, can outcompete CD28 to bind to CD80 and CD86 on APCs preventing both the activation and proliferation of T cells in the lymphnodes [22, 23]. Moreover, the highly constitutively expressed CTLA-4 on Treg cells also mediates the suppressive role of Treg cells. Anti-CTLA4 blockades significantly improve the overall survival of patients with advanced melanoma [24, 25]. Differing from CTLA4 in its mechanism of action, the PD-1/PD-L1 pathway mainly mediates T cell dysfunction in the tumor microenvironment (TME). PD-1 is induced on activated T cells through TCR and co-stimulatory signals. These effector T cells are capable of recognizing tumor neoantigens and then producing interferon-γ (INF-γ), thereby inducing the expression of PD-L1 on tumor cells and stromal cells. Ultimately, the interaction between PD-L1 and PD-1 disrupts the antitumor activity of effector T cells. This local immune escape mechanism has been termed “adaptive immune resistance” [26–29]. Sanmamed and Chen [30] have confirmed anti-PD/PD-L1 therapy as a clearest approach to the normalization of cancer immunotherapy; it can selectively repair the tumor-induced immune defect and restore immune activity in the TME without general immune activation [30]. Patients with a variety of cancer types have exhibited promising clinical benefit/risk ratio from anti-PD-1/PD-L1 therapies, resulting in FDA approval for the corresponding indications [21, 26, 30, 31].

The progress made in uncovering the biology of SCLC and its microenvironment has offered new therapeutic strategies for SCLC [32]. The high mutation burden, which produces a large number of potential tumor-specific antigens, raised hope regarding immunotherapy in SCLC [33]. In this review, we mainly illustrate the rationale behind immunotherapy for SCLC, the differences in clinical outcomes of ICBs and the underlying mechanisms in NSCLC and SCLC. Finally, we propose some strategies to improve the clinical outcome of immunotherapy for SCLC.

## Rationale of immunotherapy therapy for SCLC

Nearly 98% of SCLC patients have a smoking history [4–6]. Tobacco can exert carcinogenic effects through more than 60 chemicals that are capable of binding and mutating DNA [10]. Consequently, SCLC has a high tumor mutation burden (TMB) of 8.62 nonsynonymous or missense mutations per million base pairs among various solid tumors [7–10]. These mutations can therefore generate neoantigens (new protein or peptide sequences) that will be presented by MHC molecules and recognized by T cells [20, 34, 35]. Immunogenic neoantigens that elicit tumor-specific CD8+ T cell activities can thereby enhance the clinical benefits of immune checkpoint blockades (ICBs) [36, 37]. The TMB has been confirmed as an effective biomarker to predict the clinical outcome of ICBs in many tumors [35, 38]. Moreover, there is a similar level and distribution of TMBs between NSCLC and SCLC, and similar to in NSCLC, the TMB has been associated with the clinical outcome of ICBs in SCLC [33, 39].

Moreover, retrospective findings indicated that host immune status was strongly associated with the prognosis of SCLC. The inflammatory status of the host immune system was suggestive of a beneficial clinical outcome. Immunologic indicators associated with better prognosis included tumor-infiltrating macrophages (TIMs) and lymphocytes (TILs) [40–42], effective CD4+ T cells including Th17 cells [43], a high effector T cell (Teff) to regulatory T cell (Treg) ratio [43], a high neutrophil to lymphocyte ratio (NLR) and a high platelet to lymphocyte ratio (PLR) [44]. In addition, suppressive immune features indicating a poor prognosis of SCLC include the frequency of CD14+HLA-DR-/low myeloid-derived suppressor cells (MDSCs) [45], the C-reactive protein/albumin (CRP/Alb) ratio[46] and a higher Treg cell ratio in tumor infiltrates [47]. These clinical evidence further illustrated the significance of the immune response and the possibility of immunotherapy for SCLC.

In conclusion, the high TMB and the status of the host immune system may hold new promise for immunotherapy for SCLC. Among multiple immunotherapy strategies, ICBs have achieved great success in treating SCLC.

## Clinical outcomes of ICBs in NSCLC and SCLC

Immune checkpoint blockades, including anti-CTLA4 antibody (ipilimumab), anti-PD-1 antibodies (pembrolizumab and nivolumab), and anti-PD-L1 antibodies (atezolizumab and durvalumab), have showed remarkable and durable responses across multiple cancer types and received FDA approval [21, 26, 30, 31]. Table 1 summarizes the major clinical trials involving ICBs in NSCLC and SCLC.

**Table 1:** Comparisons of clinical outcomes of ICBs between NSCLC and SCLC

NSCLC				SCLC			
Study	Design	ORR, PFS, and OS	TRAE	Study	Design	ORR, PFS, and OS	TRAE
First-line							
NCT 00527735 [48] II R 1: 1: 1 *N* = 204	1. Placebo+Pacl+Carbo 2. Concurrent Ipi+Pacl+Carbo 3. Phased Ipi+Pacl+Carbo	irBORR, 18%, 21%, 32% mWHO-BORR, 14%, 21%, 32% Median irPFS, 4.6 m, 5.5 m, 5.7 m Median PFS, 4.2 m, 4.1 m, 5.1 m Median OS, 8.3 m, 9.7 m, 12.2 m	G3-4 6% 20% 15%	NCT 00527735 [49] II R 1: 1: 1 *N* = 130	1. Placebo+Pacl+Carbo 2. Concurrent Ipi+Pacl+Carbo 3. Phased Ipi+Pacl+Carbo	irBORR, 53%, 49%, 71% mWHO-BORR, 49%, 33%, 57% Median irPFS, 5.3 m, 5.7 m, 6.4 m Median PFS, 5.2 m, 3.9 m, 5.2 m Median OS, 9.9 m, 9.1 m,12.9 m	G3-4 9% 21% 17%
NCT01285609 [50] III R 1: 1 *N* = 749	1. Phased Placebo+Pacl+Carbo 2. Phased Ipi+Pacl+Carbo	BORR, 47%, 44% Median PFS, 5.6 m, 5.6 m Median OS, 12.4 m, 13.4 m	G3-4 35% 51%	CA184-156 [51] III R 1, 1 *N* = 954	1. Phased Placebo+Etop+Plat 2. Phased Ipi+Etop+Plat	BORR, 62%, 62% Median PFS, 4.4 m, 4.6 m Median OS, 10.9 m, 11.0 m	G3-5 11% 22%
IMpower131 [52] III R 1: 1 *N* = 578	1. Atezo+Nab-Pacl+Carbo 2. Nab-Pacl+Carbo	ORR, 49%, 41% Median PFS, 6.3 m, 5.6 m Median OS, 14.0 m, 13.9 m	G3-5 67% 58%	IMpower133 [53] III R 1: 1 *N* = 403	1. Atezo+Etop+Carbo 2. Placebo+Etop+Carbo	ORR, 60.2%, 64.4% Median PFS, 5.2 m, 4.3 m Median OS, 12.3 m, 10.3 m	G3-5 58.1% 57.6%
IMpower132 [54] III R 1: 1 *N* = 403	1. Atezo+Pem+Plat 2. Placebo+Pem+Plat	ORR, 47%, 32% Median PFS, 7.6 m, 5.2 m Median OS,18.1 m, 13.6 m	G3-5 58.1% 57.6%				
IMpower150 [55] III R 1: 1 *N* = 692	1. Atezo+Bev+Pacl+Carbo 2. Bev+Pacl+Carbo	ORR, 63.5%, 48% Median PFS, 8.3 m, 6.8 m Median OS,19.2 m, 14.7 m	G3-5 58.5% 50%				
Second-line and beyond							
CheckMate 017 [56] Squamous III R 1: 1 *N* = 272	1. Nivo 2. Docetaxel	ORR, 20%, 9% Median PFS, 3.5 m, 2.8 m Median OS, 9.2 m, 6.0 m 1-year OS, 42%, 24%	G3-4 7% 55%	CheckMate 032 [57] I/II NR, *N* = 216	1. Nivo 3 mg/kg 2. Nivo 1 mg/kg + Ipi 3 mg/kg 3. Nivo 3 mg/kg + Ipi 1 mg/kg.	ORR, 10%, 23%, 19% Median PFS, 1.4 m, 2.6 m, 1.4 m Median OS, 4·4 m, 7.7 m, 6.0 m 1-year OS, 33%, 43%, 35%	G3-4 13% 30% 19%
CheckMate 057 [58] Nonsquamous III R 1: 1 *N* = 582	1. Nivo 2. Docetaxel	ORR, 19%, 12% Median PFS,2.3 m, 4.2 m Median OS, 12.2 m, 9.4 m 1-year OS, 51%, 39%	G3-4 10% 54%	CheckMate 331 [59] III R 1: 1 *N* = 569	1. Nivo 2. Topotecan/Amrubicin	ORR, 13.7%, 16.5% Median PFS, 1.5 m, 3.8 m Median OS, 7.5 m, 8.4 m 1-year OS, 37%, 34%	G3-4 4.7% 74%
KEYNOTE-010 [60] PD-L1 ≥ 1% II/III R 1: 1: 1 *N* = 1034	1. Pembro 2 mg/kg, 2. Pembro 10 mg/kg 3. Docetaxel 75 mg/m^2^	ORR, 18%, 18%, 9% Median PFS, 3.9 m, 4.0 m, 4.0 m Median OS, 10·4 m, 12.7 m, 8.5 m 1-year OS, 43.2%, 52.3%, 34.6%	G3-5 13% 16% 35%	KEYNOTE-028 KEYNOTE-158 [61] Ib/II *N* = 83	Pembro	ORR, 19.3% Median PFS, 2.0 m Median OS, 7.7 m 1-year OS, 20.7%	G3-5 8%
POPLAR [62] II R 1: 1 *N* = 287	1. Atezo 2. Docetaxel	ORR, 15%, 15% Median PFS, 2.7 m, 3.0 m Median OS, 12.6 m, 9.7 m	G3-4 11% 39%	IFCT-1603 [63] II R 2: 1 *N* = 64	1. Atezo 2. Etop+Carbo/Topotecan	6-week ORR, 2.3%, 10% Median PFS, 1.4 m, 4.3 m Median OS, 11.4 m, 9.4 m	G3-4 4.2% ~ 35%
OAK [64] III R 1: 1 *N* = 850	1. Atezo 2. Docetaxel	ORR, 14%, 13% Median PFS, 2.8 m, 4.0 m Median OS, 13.8 m, 9.6 m	G3-4 15% 43%				
Maintenance							
PACIFIC [65] III R 2: 1 *N* = 709	1. CRT+Durva 2. CRT+Placebo	Median PFS, 17.2 m, 5.6 m Median OS, NR, 28.7 m 1-year OS, 66.3%, 55.6%	G3-4 30.5% 26.1%	CheckMate 451 III R 1: 1: 1 *N* = 810	1. Nivo 2. Nivo+Ipi 3. Placebo	Maintenance nivo/nivo+ipi did not improve OS	NA
				NCT02359019 [66] II *N* = 45	Pembro	Median PFS, 1.4 m Median OS, 9.6 m	~ 15%

*Abbreviations*: *ED* extensive-stage, *LD* limited-stage, *R* randomized, *NR* nonrandomized, *ORR* objective response rate, *PFS* progression-free survival, *OS* overall survival, *TRAE* treatment-related adverse events, *G3-5* grade 3-5, *Pacl* paclitaxel, *Carbo* carboplatin, *Ipi* ipilimumab, *Etop* etoposide, *Bev* bevacizumab, *ir* immune related, *BORR* best overall response rate, *mWHO* modified WHO, *Plat* platinum, *Nivo* nivolumab, *Pembro* pembrolizumab, *Atezo* atezolizumab

### First-line

First, Reck et al*.* conducted a randomized phase II trial to investigate ipilimumab in combination with chemotherapy in previously untreated patients with lung cancer (ED-SCLC, *n* = 130; NSCLC, *n* = 204) [48, 49]. For both the NSCLC and SCLC cohorts, phased ipilimumab with carboplatin and paclitaxel but not a concurrent regimen showed improved immune-related (ir) progression-free survival (PFS) and a numeric, though not statistically significant, increase in median overall survival (OS) over chemotherapy alone. Overall grade 3-4 (G3-4) immune-related adverse events (irAEs) were more common for phased ipilimumab. Later, the phase III CA184-156 study further evaluated the efficacy of phased ipilimumab with etoposide and platinum as the first-line regimen for ED-SCLC [51]. Disappointingly, phased ipilimumab did not significantly prolong PFS and OS over the placebo and produced more irAEs. The rate of treatment-related discontinuation was even higher (18% v 2%). Similar results were observed in patients with advanced squamous NSCLC [50]. Another phase II trial involving phased ipilimumab to first-line chemotherapy even reported a G3-5 irAE rate as high as 69.2% with 5 of 42 patients dying [67]. The unfavorable benefit to risk profile limits the first-line application of ipilimamab in lung cancer.

The addition of atezolizumab to chemotherapy as the first-line treatment significantly improved the ORR and PFS among patients with metastatic nonsquamous and squamous NSCLC in IMpower 131 and IMpower 132 [52, 54]. Among patients with metastatic nonsquamous NSCLC, atezolizumab plus bevacizumab and chemotherapy significantly improved PFS and OS in IMpower 150, which led to FDA approval of the combined regimen for the first-line treatment of nonsquamous NSCLC [55]. For ED-SCLC, the clinical efficacy and safety of atezolizumab plus chemotherapy was evaluated in IMpower133 [53]. The interim analysis of the intention-to-treat population in this phase III trial showed that the addition of atezolizumab to standard chemotherapy significantly prolonged OS and PFS over the placebo (median OS, 12.3 v 10.3 months and median PFS, 5.2 v 4.3 months) and increased the 1-year OS by 13.5% (51.7% v 38.2%). The safety profile was consistent with previous observations. In March 2019, this combined regimen was approved as a first-line therapy for ED-SCLC. Overall, atezolizumab in combination with chemotherapy as a first-line treatment could be a novel option for people with advanced NSCLC and ED-SCLC.

Pembrolizumab in combination with standard chemotherapy resulted in significantly prolonged OS and PFS compared with chemotherapy alone among patients with squamous and nonsquamous NSCLC in KEYNOTE-407 and KEYNOTE-189 [68, 69]. However, the attempt to use pembrolizumab in the first-line setting for SCLC is limited. Only the phase Ib PembroPlus trial indicated that the standard dose pembrolizumab could be safely combined with many chemotherapy regimens across 6 advanced solid tumors, including relapsed SCLC [70]. A randomized phase III trial, KEYNOTE-604, is underway to assess the clinical benefit of pembrolizumab in combination with etoposide and platinum as a first-line treatment for ES-SCLC (Table 2).

**Table 2:** Ongoing clinical trials of immune checkpoint blockades in SCLC

Study	Stage	Ph	Pts.	Design	Primary end point	NCT number	Primary completion
First-line							
KEYNOTE-604	ED	III	453	Pembro+EP v Placebo+EP	PFS, OS	NCT03066778	December 2019
REACTION	ED	II	118	Pembro+EP v EP	6-month PFS	NCT02580994	August 2020
NA	LD/ED	I	80	Pembro+EP ± RT	MTD	NCT02402920	July 31, 2023
NA	LD	II / III	506	Atezo+EP+RT v EP+RT	PFS, OS	NCT03811002	May 2024
CASPIAN	ED	III	988	Durva±Treme+EP v EP	PFS, OS	NCT030s43872	September 2019
CLOVER	LD^b^	I	300	Durva±Treme+EP	DLT; AE	NCT03509012	April 2022
PAVE	ED	II	55	Avelumab+EP	1- year PFS	NCT03568097	November 2020
Second-line and beyond							
BLOLUMA	LD/ED^a^	II	106	Nivo+Ipi	ORR	NCT03083691	March 2019
MCC-19163	LD/ED	II	41	Nivo+Ipi+Ad.p53-DC vaccine	DCR	NCT03406715	April 2020
CA209-9YT	ED	II	40	Nivo+Ipi	Teff/Treg	NCT03670056	September 2020
NA	ED	II	29	Nivo+Gemcitabine	ORR	NCT03662074	September 2020
AFT-17	LD/ED	II	98	Pembro v Topotecan	PFS	NCT02963090	May 2019
KEYNOTE PN758	ED	I/ II	84	Pembro+Pegzilarginase	AE, ORR	NCT03371979	December 2020
NA	LD/ED	II	80	Pembro+EP±RT	PD-L1	NCT02934503	October 2019
ML39728	LD/ED	II	35	Atezo	ORR	NCT03262454	December 2019
Winship3112-15	LD/ED	II	28	Durva±Treme+RT v RT	PFS, ORR	NCT02701400	January 2020
PASSION	ED	II	135	SHR-1210+Apatinib	AE, ORR	NCT03417895	March 2019
Maintenance							
STIMULI	LD	II	260	Nivo+Ipi v Obsesrvation	PFS, OS	NCT02046733	October 2019
CA209-840	ED	I/ II	21	Nivo+Ipi+RT	PFS	NCT03043599	April 2021
ACHILES	LD	II	212	Atezo v Observation	2-year OS	NCT03540420	December 2023
NA	LD	II	51	Durva+EP+RT	PFS	NCT03585998	June 2021
ADRIATIC	LD	III	600	Durva±Treme v Placebo	PFS, OS	NCT03703297	June 2021

*Abbreviations*: *ED* extensive-stage, *LD* limited-stage, *a* including NSCLC, *b* including 3 other solid tumors, *ORR* objective response rate, *PFS* progression-free survival, *OS* overall survival, *AE* adverse event, *Nivo* nivolumab, *Ipi* ipilimumab, *RT* radiotherapy, *Pem* pembrolizumab, *EP* etoposide plus platinum, *MTD* maximum tolerated dose, *Atezo* atezolizumab, *Durva* durvalumab, *Treme* tremelimumab, *DLT* dose-limiting toxicities, *NA* not available

### Second-line

As shown in Table 1, the second-line nivolumab monotherapy significantly improved ORR, PFS, and OS compared with docetaxel among patients with advanced squamous and nonsquamous NSCLC in CheckMate 017 and CheckMate 057 [56, 58]. The response rate to nivolumab monotherapy was approximately twice that of docetaxel (20% v 10%), and nivolumab extended OS by approximately 3 months over chemotherapy. For SCLC, in the nonrandomized cohort in CheckMate 032 [57], the ORR was 10% (10 of 98) and 23% (14 of 61), and the median OS was 4.4 and 7.7 months for patients receiving nivolumab 3 mg/kg and nivolumab 1 mg/kg plus ipilimumab 3 mg/kg, respectively. One-year OS was 33% and 43% for the two groups, respectively. Based on this trial, nivolumab and nivolumab plus ipilimumab were added as category 2A recommendations to the NCCN guidelines [11]. In August 2018, under accelerated approval, FDA approved nivolumab for treating patients with relapsed SCLC after the failure of platinum-based chemotherapy and one or more other lines of treatment. Unfortunately, CheckMate 331, a randomized phase III trial, demonstrated that nivolumab was inferior to topotecan or amrubicin in improving ORR, PFS, and OS among patients with relapsed SCLC [59].

Based on KEYNOTE-010, pembrolizumab was approved as a second-line treatment for advanced NSCLC patients with PD-L1 expression on ≥ 1% of tumor cells [60]. The phase Ib KEYNOTE-028 trial showed favorable efficacy and tolerable safety of pembrolizumab in treating patients with relapsed ED-SCLC and PD-L1 expression on ≥ 1% of tumor and stromal cells [71]. Further, the phase II KEYNOTE-158 trial confirmed the beneficial role of pembrolizumab in treating SCLC [72]. The latest results of KEYNOTE-028 and KEYNOTE-158 from 2019 from the American Association for Cancer Research (AACR) showed that pembrolizumab produced a durable response with tolerable toxicity for advanced SCLC patients after ≥ 2 lines of prior therapy. The ORR was 19.6% (16 of 83), with 2 patients having a complete response (CR) and 14 having a partial response (PR). More than half (9 of 16) had a response duration of ≥ 18 months. The median PFS was 2.0 months, and the median OS was 7.7 months, with a 1-year OS rate of 20.7%. The toxicity was manageable, with a G3-5 AE incidence of 9% [61]. Despite the encouraging results of single-arm studies, large randomized controlled studies are needed.

Atezolizumab also significantly improved OS by 3 to 4 months over docetaxel in patients with previously treated NSCLC in POPLAR and OAK [62, 64]. Unfortunately, the randomized phase II IFCT-1603 trial indicated that atezolizumab was not superior to chemotherapy as a second-line treatment in SCLC [63].

### Maintenance

Durvalumab, an anti-PD-L1 antibody, has significantly prolonged PFS by more than twofold compared with the placebo (17.2 v 5.6 months, HR = 0.51) among patients with unresectable stage III NSCLC without disease progression after concurrent chemoradiotherapy in PACIFIC [65]. For patients with SCLC, the clinical outcome of ICBs as a maintenance regimen is quite dissatisfactory. A single-group, phase II study showed that maintenance pembrolizumab did not prolong OS compared with historical chemotherapy after first-line chemotherapy in patients with ED-SCLC [66]. In November 2018, Bristol-Myers Squibb announced that the phase III CheckMate 451 trial did not meet the primary endpoint of OS with nivolumab and nivolumab plus ipilimumab versus the placebo as maintenance therapy in patients with ED-SCLC.

In conclusion, anti-PD-1/PD-L1 blockades have revolutionized the standard treatment for NSCLC across the first-line, second-line, and maintenance settings based on multiple large randomized controlled trials (Table 1). However, for SCLC, despite moderate benefit in phase I/II trials, ICBs did not outcompete traditional chemotherapy in large randomized trials. Only atezolizumab in combination with chemotherapy conferred a survival benefit in CheckMate 331. Table 2 lists the ongoing trials involving ICBs or combination regimens in SCLC.

## Potential mechanisms underlying discrepant outcomes of ICBs between SCLC and NSCLC

SCLC, with a high TMB similar to that of NSCLC, is theoretically likely to respond to immunotherapy. However, the clinical outcome of ICBs in SCLC seems far less effective than in NSCLC. There is a need to define the underlying differences in immune regulation patterns between SCLC and NSCLC to further guide immunotherapy for SCLC.

### Low PD-L1 expression in SCLC

PD-L1 expression reflects pre-existing antitumor immunity in the TME and has been associated with a clinical benefit from anti-PD-1/PD-L1 blockade across multiple cancer types, including NSCLC [73–75]. PD-L1 expression in NSCLC is approximately 50–70% according to previous reports [55, 56, 58, 62, 64, 68, 69, 76]. In stark contrast to NSCLC, PD-L1 expression in SCLC has been reported to be relatively low in most studies, as listed in Table 3. Most studies showed less than 50% PD-L1 expression in SCLC. The reason for the disparity in PD-L1 expression in these findings is not well understood and may be explained by the differences in staining antibodies, scoring algorithms, tissue biopsy types (archival or fresh), and detection platforms (Table 3). Notably, substantial PD-L1 expression occurs on stroma cells, including tumor-infiltrating immune cells (TILs and TIMs), in SCLC and less in tumor cells. Some of these studies demonstrated a positive correlation between PD-L1 expression on TICs and a favorable clinical outcome for SCLC patients [41, 66, 77, 80, 81]. Emerging data indicated that infiltrating stroma cells, such as dendritic cells and macrophages, could have protumorigenic functions by shaping antitumor immunity and the response to immunotherapy [84–86]. Perhaps PD-L1 expression in immune cells may play a exceptional role in the pathophysiological process in SCLC. Overall, the relatively low PD-L1 expression may be at least one reason that the efficacy of ICBs in SCLC is not as good as that in NSCLC.

**Table 3:** PD-L1 expression in SCLC

Author	Patients^c^	IHC assay	Biopsy types	TC PD-L1 cutoff	Cell type	PD-L1 expression
Antonia et al*.* [57]	146	28-8/Dako	Archival or fresh	1% 5%	Tumor cell	16.4% 4.8%
Ott et al. [71]	145	22C3/Dako	Archival or fresh	1%	Tumor and Stromal cell	31.7%
Hyun et al. [72]	92	22C3/Dako	Archival or fresh	1%	Tumor and stromal cell	45.7%
Inamura et al. [77]	74	E1 L3N/CST	Archival	5%	Tumor cell	18.9%
Yasuda et al. [78]	39	22C3/Dako	Archival	1%	Tumor cell	2.5%
Pujol et al. [63]	53	SP142/Ventana	Archival	1%	Tumor and stromal cell	2%
Gadgeel et al. [66]	30 20	22C3/Dako	Archival	1%	Tumor cell Stromal cell	10% 40%
Schultheis et al. [79].	94^d^	5H1/Chen’s lab E1 L3N/CST	Archival	1%	Tumor cell Stromal cell	0.0% TIMs, 18.5% TILs, 48%
Berghoff et al. [41]	32	NA/Dako	Archival	5%	Tumor cell Stromal cell	34.4% TIMs, 28.1% TILs, 25.0%
Yu et al. [80]	194^e^	SP142/Ventana 28-8/Dako	Archival	1%	Tumor cell Stromal cell	16.5% TILs, 56.2%, 44.8%
Kim et al. [81]	120	MAB1561/ R&D system	Archival	1%	Tumor cell Stromal cell	14.2% TILs, 23.3%
Komiya et al [82]	99	EPR1161/Abcam	Archival	5%	Tumor cell	82.8%
Ishii et al. [83]	102	NA/Abcam	Archival	5%	Tumor cell	71.6%

*Abbreviations*: *c* patients who are PD-L1 evaluable, *d* small cell carcinomas 94 patients (SCLC 61; extrapulmonary 33), *e* total 194 patients, LD-SCLC 98,ED-SCLC 96, *IHC* immunohistochemistry, *TC* tumor cell, *CST* cell signaling technology, *TIMs* tumor infiltration macrophage, *TILs* tumor-infiltrating lymphocytes, *NA* not available*

### Downregulation of MHC molecules in SCLC

The downregulation of MHC molecules is an immune escape mechanism. In contrast to NSCLC cells, which readily express MHC class I molecules, most SCLC cell lines and tissues showed marked deficient expression of MHC class I molecules, thus directly preventing tumor cells from presenting neoantigens to CD8+ T cells in the lymph nodes and inhibiting CTL recognition in the TME [87, 88]. Additionally, due to the lack of IFN-γ inducible expression of class II transactivator (cIITA), MHC class II molecule expression is absent in SCLC cells and significantly lower in SCLC TILs compared with in NSCLC, resulting in reduced presentation of tumor neoantigens to CD4+ T cells. In addition, mainly induced by IFN-γ-producing T cells, MHC class II molecules also reflect a less immunogenic environment in SCLC compared with NSCLC [89–91].

### Immunosuppression induced by SCLC cells

The interaction between SCLC cells and the immune system seems to be more complicated than previously thought. Tumors may destroy the host immune system in a variety of ways. The functions of the immune system, including lymphocyte reactivity to lectins and cytokines and the production of cytokines, were even more impaired in patients with SCLC compared with patients with NSCLC. The peripheral blood lymphocytes (PBLs) of SCLC patients showed significantly lower proliferative responses to phytohemagglutinin and human recombinant interleukin 2 (IL-2) than those of the NSCLC and noncancer groups. The ability of PBL to produce lymphokines (IL-2 and macrophage-activating factor) was evidently impaired in the SCLC group but not in the NSCLC group [92]. In contrast to NSCLC, the suppression of cytokine secretion in SCLC was dependent on tumor load and could be improved upon the reduction of the tumor load [93]. In addition, Wang et al. found that IL-15 secreted by SCLC cells could contribute to local and systemic immune escape and poor prognosis by inhibiting CD4+ T cell proliferation and supporting Treg cell induction [47]. Moreover, CD47 mainly functions to inhibit the activation and phagocytosis of macrophages through CD47/SIRPα signaling[94]. The remarkable upregulation of CD47 on the SCLC cell surface may be another important immune escape mechanism by inhibiting the activation and phagocytosis of macrophages. Preliminary experiments suggested that blocking CD47 induced macrophage-mediated phagocytosis in SCLC cell lines and mouse xenograft models [95]. Dysregulation of the Fas/FasL signaling pathway in tumors by reducing Fas expression and increasing FasL expression could participate in tumor development and immune escape [96, 97]. Fas expression is markedly decreased and even completely lost in lung tumors. However, FasL expression is decreased in most NSCLCs but is upregulated in 91% of SCLCs. FasL overexpression in the context of Fas downregulation in SCLC confers the ability on SCLC cells of inducing paracrine killing of Fas-expressing cytotoxic T cells and inhibiting self-apoptosis. These results indicated that the heavy tumor burden in SCLC makes the immune system less functional than in NSCLC through IL-15 secretion and the CD47/SIRPα and Fas/FasL pathways.

### Autocrine and paracrine regulation in SCLC

A distinguishing feature of SCLC is the substantial autocrine and paracrine stimulation by growth factor receptors and chemokine receptors. Bombesin/gastrin-releasing peptide (BN/GRP) is a relevant growth factor in SCLC, and its receptor in overexpressed in SCLC [98]. Compared with NSCLC, which does not secrete autocrine growth factor granulocyte chemotactic protein (GCP-2), SCLC constantly secretes GCP-2 and expresses its receptors, CXCR1 and CXCR2 [99]. Stem cell factor (SCF) and its receptor c-kit, expressed in 40–70% SCLC specimens, are important regulators of SCLC viability [100]. In contrast to NSCLC cell lines, SCLC cell lines do not express intercellular adhesion molecule-1 (ICAM-1), which is involved in the interaction with lymphokine-activated killer cells [101]. IL-8 acts as an autocrine and/or paracrine growth factor for lung cancer, mediated by CXCR1 and CXCR2 receptors on the tumor cell surface. However, NSCLC cells produce much higher levels of IL-8 than SCLC cells. As a result, IL-8 may attract neutrophils and induce the immune response in NSCLC but mainly promote autocrine growth in SCLC [102, 103]. Lopez-Gonzalez et al. found that NSCLC and SCLC cell lines had diverse expression levels of TGF-β and its receptor [104]. NSCLC synthesizes both TGF-β isoforms and TGF-β RII. In stark contrast, malignant transformation in SCLC alters the synthesis of TGF-β isoforms and TGF-β RII, thereby avoiding autocrine and paracrine growth inhibition by TGF-β in SCLC cells. Another study found that four to eight SCLC cell lines could constantly secrete biologically active TGF-β1 to suppress IL-2-dependent T cell growth, and a specific anti-TGF-β1 antibody blocked the immunosuppressive activity induced by the SCLC cell lines [105]. These studies suggested that SCLC cells could markedly promote self-growth via autocrine and paracrine regulation but had less immune stimulatory function in adjacent immune cells compared with NSCLC cells.

These comparative analyses of immune regulation patterns between SCLC and NSCLC partially explain the immunodeficiency observed in patients with SCLC and the worse immune response in SCLC patients. Detailed investigations are essential to determine the exact immune escape mechanisms in SCLC to support the development of immunotherapy for SCLC.

## Future of immunotherapy for SCLC

As demonstrated above, there are many differences underlying immune regulation patterns between NSCLC and SCLC. We note that several potential strategies could possibly improve the clinical outcome of immunotherapy for SCLC.

### Chemotherapy and radiotherapy prior to ICBs

The intrinsic nature of ED-SCLC is so aggressive that a great proportion of patients dropped out or discontinued without completing the whole treatment course [51]. As illustrated in part 3, SCLC cells can induce immunosuppression and promote autocrine and paracrine regulation. Immune suppression seems to be associated with tumor load and could improve upon the reduction of the tumor load [93]. Chemotherapy and radiotherapy can not only reduce the heavy tumor burden of SCLC and further reinvigorate immune function but can also elevate PD-L1 expression and tumor antigen presentation by MHC molecules, thus favoring subsequent immunotherapy [106, 107]. We speculate that chemotherapy and radiotherapy prior to ICBs could be effective in improving the clinical outcome of immunotherapy for SCLC.

### Combination of anti-angiogenesis and ICBs

Angiogenesis is one of the hallmarks of cancer [108]. Anlotinib, an oral anti-angiogenesis tyrosine multikinase inhibitor, is an approved third-line or later therapy for advanced NSCLC according to the Chinese Food and Drug Administration (CFDA), based on the ALTER 0303 study [109]. ALTER 1202, a phase II trial, has also demonstrated that anlotinib significantly improves OS as a third-line or later treatment [110]. Combination therapy with antiangiogenic therapy is a new paradigm in the treatment of advanced-stage malignancies via normalizing tumor vasculature and increasing drug delivery [111, 112]. Vascular endothelial growth factor (VEGF), the major regulator in tumor angiogenesis, plays an important role in immune modulation by blocking dendritic cell (DC) differentiation, reducing T cell infiltration, inducing inhibitory immune cells (Tregs and MDSCs) and inhibiting T cell development [112, 113]. Theoretically, combining immunotherapy with antiangiogenic treatment may have a synergistic effect, enhance the efficacy of both. Atezolizumab in combination with bevacizumab and chemotherapy significantly improved OS among patients with metastatic nonsquamous NSCLC in IMpower 150 [55]. The combination of ICBs with antiangiogenic therapy could also be promising in SCLC.

### Biomarker selection

Compared with NSCLC, PD-L1 expression is relatively low in SCLC. In NSCLC, multiple clinical trials have defined PD-L1 as a biomarker for pembrolizumab, and the FDA-approved IHC assay utilized cutoffs of a 50% tumor proportion score for first-line and 1% for second-line pembrolizumab therapy [114]. Although PD-L1 expression was associated with a survival benefit in SCLC as indicated in early phase studies [66, 71, 72], it is not a perfect biomarker in SCLC. TMB, another biomarker of immunotherapy, could also predict the clinical outcome of ICBs based on exploratory analyses of CheckMate 026 [39] and CheckMate 227 [76] in NSCLC. In SCLC, Hellmann et al. retrospectively evaluated 211 TMB-evaluable patients from the total nonrandomized and randomized cohorts of CheckMate 032 [33]. For patients receiving nivolumab monotherapy and nivolumab plus ipilizumab, the ORR was higher in patients with a high TMB (21.3% and 46.2%) than in patients with a medium (6.8% and 16.0%) or low (4.8% and 22.2%) TMB. The OS for SCLC patients with a high TMB treated with nivolumab plus ipilimumab (22 months) was nearly three times that (6–8 months) achieved by the historical topotecan chemotherapy [16, 17]. Additionally, there was no association between PD-L1 expression and TMB in lung cancer [33, 39, 115]. Patients receiving nivolumab with a high level of these two biomarkers had a higher ORR than those with only one of these factors in CheckMate 026 [39]. Up to now, there are no exact biomarkers for immunotherapy in SCLC, TMB seemed to be more important than PD-L1 expression and perhaps the combination of the two biomarkers will be more valuable for selecting patients who will benefit from ICBs.

## Conclusion

SCLC is an aggressive cancer type with poor prognosis. Limitations in the current treatment choices for SCLC give impetus to the search for novel therapeutic approaches including immunotherapy. Despite the similar level and distribution of the TMB between NSCLC and SCLC, the clinical efficacy of ICBs for SCLC is far less pronounced than that for NSCLC. Most studies concerning ICBs in SCLC failed to elicit better clinical outcomes than traditional chemotherapy. The potential mechanisms involved may be (1) low PD-L1 expression in SCLC, (2) downregulation of MHC molecules in SCLC, (3) immunosuppression induced by SCLC cells, and (4) autocrine and paracrine regulation in SCLC. Nevertheless, ICBs also brought about promising clinical benefits in SCLC. Atezolizumab in combination with etoposide and carboplatin prolonged OS by 2 months over chemotherapy alone and has recently received FDA approval as the first-line treatment for SCLC.

How to improve the clinical efficacy of immunotherapy for SCLC is an essential issue. Based on the dysregulation of the immune system in SCLC, we expect that chemotherapy and radiotherapy prior to immunotherapy could be more effective in improving the clinical outcome of ICBs in patients with SCLC. The combination of ICBs with antiangiogenic therapy may be a novel option for SCLC. Last, TMB seemed to be a more valuable biomarker for the efficacy of ICBs in SCLC, and the combination of TMB and PD-L1 may be promising for selecting patients who benefit from immunotherapy in SCLC.

## Data Availability

The dataset supporting the conclusions of this article is included within the article.

## Abbreviations

AACR: American Association for Cancer Research
AE: Adverse event
CFDA: Chinese Food and Drug Administration
CTLA4: Cytotoxic T-lymphocyte protein-4
ES-SCLC: Extensive-stage SCLC
FDA: US Food and Drug Administration
G3-4: Grade 3-4
GCP-2: Granulocyte chemotactic protein
ICAM: Intercellular adhesion molecule-1
ICB: Immune checkpoint blockade
INF-γ: Interferon-γ
ir: Immune-related
LS-SCLC: Limited-stage SCLC
MHC: Major histocompability complex
NSCLC: Non-small cell lung cancer
ORR: Overall response rate
OS: Overall survival
PD-1: Programmed cell death protein-1
PD-L1: Programmed cell death ligand-1
PFS: Progression-free survival
SCF: Stem cell factor
SCLC: Small cell lung cancer
TIL: Tumor infiltration lymphocyte
TIM: Tumor infiltration macrophage
TMB: Tumor mutation burden
TME: Tumor mutation burden
